# Revealing the Characteristics of Glucose- and Lactate-Based Chain Elongation for Caproate Production by *Caproicibacterium lactatifermentans* through Transcriptomic, Bioenergetic, and Regulatory Analyses

**DOI:** 10.1128/msystems.00534-22

**Published:** 2022-09-08

**Authors:** Huilin Wang, Weicheng Zhou, Jiangjing Gao, Cong Ren, Yan Xu

**Affiliations:** a Lab of Brewing Microbiology and Applied Enzymology, Key Laboratory of Industrial Biotechnology of Ministry of Education, Jiangnan University, Wuxi, China; b The Center for Solid-state Fermentation Engineering of Anhui Province, Bozhou, China; University of California San Diego

**Keywords:** caproate-producing bacterium, *Caproicibacterium lactatifermentans*, lactate utilization, transcriptomics, transcriptional regulator

## Abstract

Caproate, an important medium-chain fatty acid, can only be synthesized by limited bacterial species by using ethanol, lactate, or certain saccharides. *Caproicibacterium lactatifermentans* is a promising caproate producer due to its glucose and lactate utilization capabilities. However, the global cellular responses of this bacterium to different carbon sources were not well understood. Here, *C. lactatifermentans* showed robust growth on glucose but more active caproate synthesis on lactate. Comparative transcriptome revealed that the genes involved in reverse β-oxidation for caproate synthesis and V-type ATPase-dependent ATP generation were upregulated under lactate condition, while several genes responsible for biomass synthesis were upregulated under glucose condition. Based on metabolic pathway reconstructions and bioenergetics analysis, the biomass accumulation on glucose condition may be supported by sufficient supplies of ATP and metabolite intermediates via glycolysis. In contrast, the ATP yield per glucose equivalent from lactate conversion into caproate was only 20% of that from glucose. Thus, the upregulation of the reverse β-oxidation genes may be essential for cell survival under lactate conditions. Furthermore, the remarkably decreased lactate utilization was observed after glucose acclimatization, indicating the negative modulation of lactate utilization by glucose metabolism. Based on the cotranscription of the lactate utilization repressor gene *lldR* with sugar-specific PTS genes and the opposite expression patterns of *lldR* and lactate utilization genes, a novel regulatory mechanism of glucose-repressed lactate utilization mediated via *lldR* was proposed. The results of this study suggested the molecular mechanism underlying differential physiologic and metabolic characteristics of *C. lactatifermentans* grown on glucose and lactate.

**IMPORTANCE** *Caproicibacterium lactatifermentans* is a unique and robust caproate-producing bacterium in the family Oscillospiraceae due to its lactate utilization capability, whereas its close relatives such as *Caproicibacterium amylolyticum*, Caproiciproducens galactitolivorans, and *Caproicibacter fermentans* cannot utilize lactate but produce lactate as the main fermentation end product. Moreover, *C. lactatifermentans* can also utilize several saccharides such as glucose and maltose. Although the metabolic versatility of the bacterium makes it to be a promising industrial caproate producer, the cellular responses of *C. lactatifermentans* to different carbon sources were unknown. Here, the molecular mechanisms of biomass synthesis supported by glucose utilization and the cell survival supported by lactate utilization were revealed. A novel insight into the regulatory machinery in which glucose negatively regulates lactate utilization was proposed. This study provides a valuable basis to control and optimize caproate production, which will contribute to achieving a circular economy and environmental sustainability.

## INTRODUCTION

The increasing consumption of petroleum and coal has been causing increasing environmental, ecological, and social issues. To reduce petroleum and coal usage and achieve environmental sustainability, the transformation of renewable resources into biofuels and biochemicals has gathered much attention. Anaerobic fermentation is an eco-friendly approach to convert renewable organic feedstocks into biogas, bioalcohols, and fatty acids (1). Among these products, medium-chain fatty acids (MCFAs), with 6 to 12 carbon atoms, have high added value and wide applications in food, agriculture, and various industries. For instance, caproate can be used as a food additive in the food industry, an antimicrobial agent in the pharmaceutical industry, and a precursor of lubricants in the chemical industry (2). Furthermore, MCFAs have higher market value and are easier to be separated from fermentation broths, and thus they are considered more economical than short-chain fatty acids (SCFAs), such as acetate and butyrate (3–5).

The cost-effective and eco-friendly renewable feedstocks, such as lignocellulose, food wastes and wastewater, are promising alternatives to petrochemical- and vegetal-origin feedstocks for MCFA production (6, 7). The indigenous substances or hydrolysis products of these renewable feedstocks, such as alcohols, lactate, and sugars, can be used as the substrates for MCFA production (8). Because of the complex substrate composition in nonsterile renewable feedstocks, microbial consortia rather than monocultures are more frequently used as biocatalysts to convert these renewable feedstocks into MCFAs (9–11). For instance, lactate fermentation is usually inevitable in nonsterile fermentation because sugars, the readily available substrates in many feedstocks (lignocellulosic hydrolysate, dairy, and liquor-making wastewater), are usually first utilized by lactic acid bacteria, and the produced lactate is then used as a carbon source for MCFA-producing bacteria (12–14). Therefore, in these cases, stable and efficient MCFA production relies on metabolic collaboration between lactic acid bacteria and MCFA-producing bacteria. Nevertheless, coordinating multispecies with different divisions of labor within a microbiome is still challenging (15–17). One way to cope with this challenge is to establish a two-stage fermentation system that separates lactate-producing bacteria and MCFA-producing bacteria into two different bioreactors (18). However, using two-stage fermentation systems may reduce substrate utilization efficiency and increase processing cost. Instead, it would be ideal if sugars and lactate could be coutilized by a dominant species within a microbiome for MCFA production.

So far, microbes with lactate and sugar coutilization capability have been reported to distribute in several genera, including *Caproicibacterium*, *Clostridium*, *Acetobacterium*, *Megasphaera*, *Anaerobutyricum*, Pseudomonas, and *Corynebacterium* (19–27). Although the production of caproate, a six-carbon MCFA, from sugars (fructose, glucose, and xylose) or lactate has been extensively reported (28–34), the coutilization of sugars and lactate to produce caproate as the major fermentation end product is only limited to Megasphaera hexanoica and *Caproicibacterium lactatifermentans* (19, 25). Except for these two species, other sugar- and lactate-utilizing bacteria either produce short-chain fatty acids (SCFAs) as main end products or exhibit a preference for a certain carbon source (35). For instance, Clostridium tyrobutyricum can simultaneously utilize glucose and lactate to produce butyrate as the dominant product (20). *C. lactatifermentans*, a recently validly published bacterium from Chinese liquor (*Baijiu*) anaerobic fermentation system, is the only lactate-utilizing caproate-producing species within the family Oscillospiraceae as of 2022 though this family is usually associated with the production of SCFAs and MCFAs from diverse carbohydrates (36). Furthermore, *C. lactatifermentans* has been demonstrated as a keystone species in natural MCFA-producing habitats and artificial MCFA fermentation systems (19), suggesting that this species has the potential to dominate in multispecies MCFA-producing systems and thus can be a promising candidate for biotransformation renewable feedstocks into MCFA. Our previous study has shown that *C. lactatifermentans* has better growth on glucose but a higher caproate production rate on lactate (19). This phenomenon seems contradictory because caproate synthesis is the primary metabolism, and thus biomass accumulation should have been closely linked to the caproate synthesis. The genetic mechanism underlying the unexpecting phenomenon remains to be explored.

In this study, we comprehensively demonstrated the transcriptomic changes of *C. lactatifermentans* grown on glucose and lactate. Stoichiometric and thermodynamic models for glucose- and lactate-based chain elongation were established to systemically evaluate the energy metabolism under these two culturing conditions. An interactive regulation mechanism between glucose utilization and lactate utilization was proposed. Furthermore, a deeper understanding of carbon and energy metabolism in *C. lactatifermentans* will benefit to develop this bacterium as a novel chassis for MCFA production.

## RESULTS

### Global transcriptional responses to glucose and lactate.

**FIG 1:**
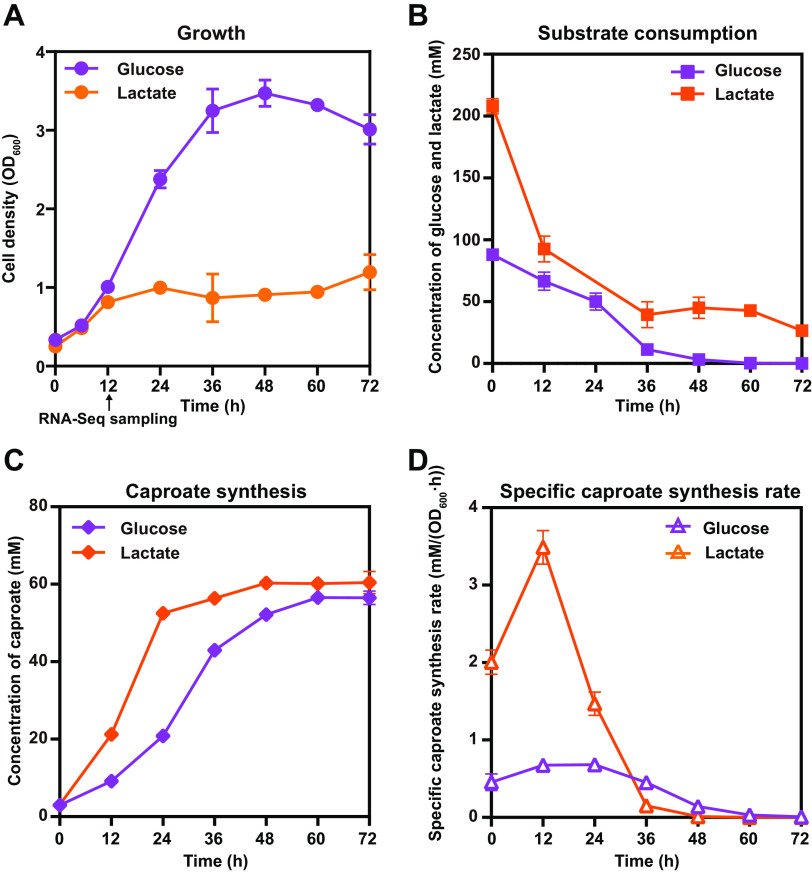
Differences in (A) cell growth, (B) substrate consumption, and (C and D) caproate production of *C. lactatifermentans* using glucose or lactate as a carbon source. The initial concentrations of glucose and lactate were approximately 16 g · L^−1^ and 19 g · L^−1^, respectively. When considering carbon availability, one mole of glucose is equivalent to two moles of lactate. Acetate was supplemented in the initial medium, and the molar ratios of glucose to acetate and lactate to acetate were around 1.5:1 and 3:1, respectively. The error bars represent the standard deviation of triplicates.

*Caproicibacterium lactatifermentans* produces caproate and butyrate as the main fermentation end products when glucose or lactate was used as carbon source (19, 37). When glucose or lactate was separately supplemented as a carbon source, the maximum biomass (OD_600_) of *C. lactatifermentans* by using glucose was almost three times higher than that by using lactate (Fig. 1A), whereas the glucose equivalent of consumed lactate was nearly the same as the consumed glucose at around 90 mM (Fig. 1B). Finally, a similar amount of around 60 mM caproate was produced (Fig. 1C). The specific caproate synthesis rate reached its maximum level at 12 h regardless of using glucose or lactate as a carbon source. However, the maximum specific caproate synthesis rate under the lactate condition was five times that under the glucose condition (Fig. 1D). These phenomena indicated that glucose promoted cell growth, whereas lactate promoted faster caproate production.

**FIG 2:**
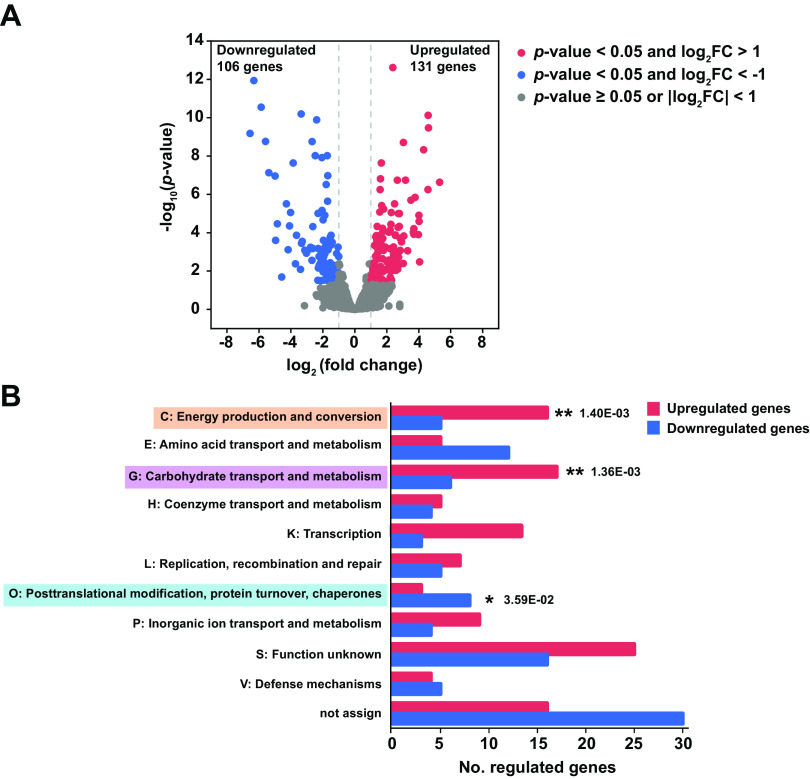
Transcriptomic responses of *C. lactatifermentans* using glucose or lactate as a carbon source. (A) Volcano plot of RNA-seq data showing the significantly differentially expressed genes of *C. lactatifermentans* grown on glucose or lactate. Genes that have higher expression levels under lactate conditions than glucose conditions are defined as upregulated genes. (B) Enrichment analysis of differentially expressed genes based on clusters of orthologous groups (COG). Only those COGs containing more than five genes under any culture condition were shown. The significant enrichment COG categories were marked with asterisks (*, *P < *0.05; **, *P < *0.01).

The carbon source metabolism is closely related to diverse physical and metabolic activities in cells (38). To determine how *C. lactatifermentans* responds to different carbon sources, RNA-seq was used to compare the global transcriptional differences between the glucose and lactate conditions. We found that the transcription of 237 genes statistically demonstrated differences between these two different culturing conditions, accounting for around 12% of 1,936 detected genes. Among these 237 differentially expressed genes, 131 genes were upregulated, and 106 genes were downregulated under the lactate condition (Fig. 2A). To validate the results of RNA-Seq, 16 significantly differentially expressed genes in central metabolic pathways were selected and their expressions under the glucose and lactate conditions were quantified by quantitative reverse transcription-PCR (qRT-PCR). The qRT-PCR results are shown in Fig. S1. The fold changes calculated from RNA-seq data showed high correlation with those from the qRT-PCR data (Fig. S2), suggesting that the comparative analysis based on RNA-seq data were reliable. To identify the general biological function of these differentially expressed genes, the 237 differentially expressed genes were classified into 17 clusters of orthologous groups (COGs) by using the eggNOG database. The upregulated genes under the lactate condition were significantly enriched in energy production and conversion (category C) as well as carbohydrate transport and metabolism (category G), whereas the downregulated genes were significantly enriched in posttranslational modification, turnover, and chaperones (category O) (Fig. 2B). Notably, the upregulated genes belonging to carbohydrate transport and metabolism (category G) were mainly involved in nonglucose carbohydrate metabolism. For instance, the most upregulated gene in category G *malQ* (GJQ69_02680), encoding a 4-alpha-glucanotransferase responsible for starch and maltose utilization, showed a 9.8-fold higher expression level under the lactate condition than the glucose condition (Data set S1). Genes *glgP* (GJQ69_02685) and *glgB* (GJQ69_01455) related to glycogen metabolism, showed 7.6- and 2.8-fold higher expression levels under lactate condition, respectively (Data set S1). In addition, *nagA* (GJQ69_02445) encoding GlcNAc-6-P deacetylase and *nagB* (GJQ69_02440) encoding GlcN-6-P deaminase, showed slightly increased expression (Data set S1). GlcNAc-6-P deacetylase is required for catabolism of *N*-acetylglucosamine (GlcNAc) into acetate, and GlcN-6-P deaminase is required for the catabolism of GlcNAc to fructose 6-phosphate (39). Therefore, the upregulated expression of *nagA* and *nagB* may provide acetate and fructose 6-phosphate for cells grown on lactate. Moreover, *cstA* (GJQ69_02630), encoding the carbon starvation protein and responding to starvation in Escherichia coli (40), was significantly upregulated (39.6-fold change) with a higher expression level (average transcriptional level [transcripts per million, TPM] of 2,753) under the lactate condition (Data set S1). Considering the lower growth and the upregulation of *cstA*, it was speculated that *C. lactatifermentans* suffered starvation stress grown on lactate as the dominant carbon source. A previous study demonstrated that the robust growth of Escherichia coli was closely linked to many genes response for carbohydrate transport and metabolism (38). Here, these upregulated genes in carbohydrate transport and metabolism might be essential for the survival of *C. lactatifermentans* under starvation stress.

10.1128/msystems.00534-22.4 FIG S1 Quantification of expression levels of significantly differentially expressed genes by qRT-PCR. (A–E) Significantly higher expressed genes under glucose condition; (F–P) Significantly higher expressed genes under lactate condition. The relative expression levels were normalized to the expression level of the 16S rRNA gene. The relative expression level of the control sample was set as 1.0. Data were reported as the mean ± SD of the results from three independent experiments. Download FIG S1, EPS file, 1.5 MB. Copyright © 2022 Wang et al. 2022 Wang et al. https://creativecommons.org/licenses/by/4.0/ This content is distributed under the terms of the Creative Commons Attribution 4.0 International license.

10.1128/msystems.00534-22.5 FIG S2 The correlation analysis for the differential expression levels between RNA-Seq and qRT-PCR. The genes used for this analysis are indicated in Fig. S1. Download FIG S2, EPS file, 0.9 MB. Copyright © 2022 Wang et al. 2022 Wang et al. https://creativecommons.org/licenses/by/4.0/ This content is distributed under the terms of the Creative Commons Attribution 4.0 International license.

### The effects of glucose and lactate on the expression of genes responsible for carbon metabolism, energy metabolism, and biomass synthesis.

**FIG 3:**
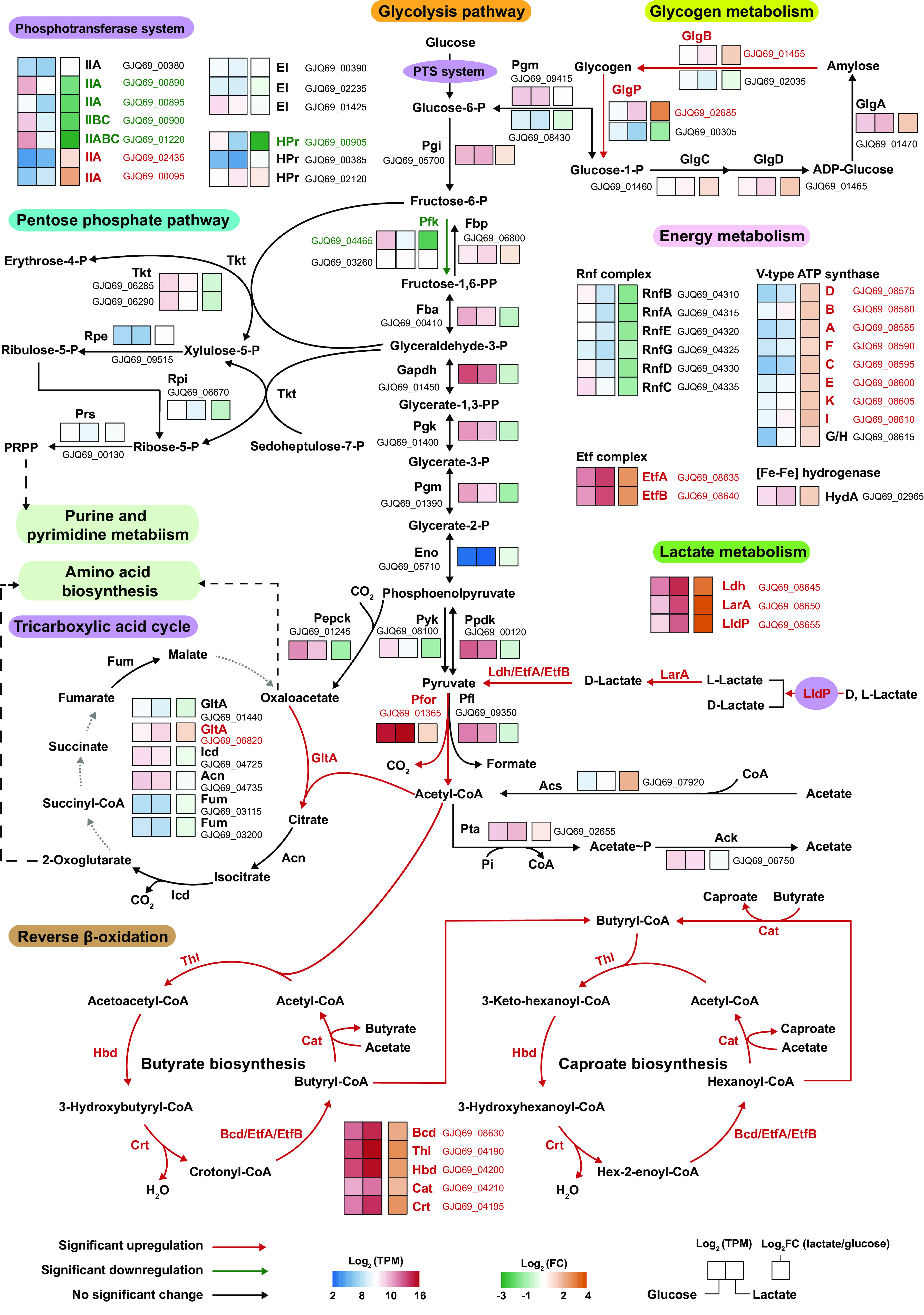
Differential gene expression profiles analysis in carbon metabolic pathways and energy production-related pathways of *C. lactatifermentans* based on transcriptional analysis. The significantly upregulated genes under lactate conditions are shown in red, the significantly downregulated genes under lactate conditions are shown in green, and nonsignificantly changed genes were shown in black. The transcriptional expression levels and changes of lactate versus glucose were normalized as log_2_ (TPM) and log_2_ (FC), respectively. PTS, phosphotransferase system; HPr, phosphoryl carrier protein; EI, PTS enzyme I; Pgi, glucose-6-phosphate isomerase; Pfk, 6-phosphofructokinase; Fbp, fructose-1,6-bisphosphatase; Fba, fructose-bisphosphate aldolase; Gapdh, glyceraldehyde-3-phosphate dehydrogenase; Pgk, phosphoglycerate kinase; Pgm, 2,3-bisphosphoglycerate-independent phosphoglycerate mutase; Eno, enolase; Pyk, pyruvate kinase; Ppdk, pyruvate phosphate dikinase; Pfl, pyruvate formate-lyase; Pfor, pyruvate: ferredoxin oxidoreductase; Pta, phosphotransacetylase; Ack, acetate kinase; Acs, acetyl-coenzyme A synthetase; Tkt, transketolase; Rpe, ribulose-phosphate 3-epimerase; Rpi, ribose-5-phosphate isomerase; Prs, ribose-phosphate pyrophosphokinase; Pepck, phosphoenolpyruvate carboxykinase; GltA, citrate synthase; Acn, aconitate hydratase; Icd, isocitrate dehydrogenase; Fum, fumarate hydratase; LldP, l-lactate permease; LarA, lactate racemase; Ldh, lactate dehydrogenase; Thl, thiolase; Hbd, 3-hydroxybutyryl-CoA dehydrogenase; Crt, enoyl-CoA hydratase; Bcd, butyryl-CoA dehydrogenase; EtfA, electron transfer flavoprotein subunit alpha; EtfB, electron transfer flavoprotein subunit beta; Cat, butyryl-CoA:acetate CoA transferase; HydA, hydrogenase; GlgA, glycogen synthase; GlgB, 1,4-alpha-glucan branching enzyme; GlgC, glucose-1-phosphate adenylyltransferase; GlgD, glucose-1-phosphate adenylyltransferase; GlgP, glycogen phosphorylase; PRPP, 5-phospho-alpha-d-ribose 1-diphosphate. A full list of gene expression data can be found in the supplemental file: Data set S1.

The enzyme II (EII) components of sugar phosphotransferase system (PTS)-encoding genes (GJQ69_00890, GJQ69_00895, GJQ69_00900, and GJQ69_01220) and a phosphocarrier protein (HPr)-encoding gene (GJQ69_00905) were significantly upregulated with glucose as carbon source, suggesting that glucose was taken up by PTS rather than ATP-binding cassette (ABC) transporter in *C. lactatifermentans* (Fig. 3). Although the gene GJQ69_00900 was annotated as fructose PTS IIBC by NCBI prokaryotic genome annotation pipeline (PGAP), gene GJQ69_00900 is more likely to be mainly responsible for glucose uptake in *C. lactatifermentans*. Nevertheless, except for the PTS system, most glycolytic genes showed similar expression levels under glucose and lactate conditions (Fig. 3), implying that gluconeogenesis may be activated when lactate was utilized. This speculation was supported by the considerable expressions of a gluconeogenesis-specific gene (*fbp*, GJQ69_06800, TPM of 1,328) encoding fructose-1,6-bisphosphatase and a gene (*ppdk*, GJQ69_00120, TPM of 7,046) encoding pyruvate phosphate dikinase under the lactate condition (Fig. 3). Gluconeogenesis may be employed to synthesize metabolites that were required for the pentose-phosphate pathway (PPP) as well as the incomplete TCA cycle (Fig. 3). Similar to the expression pattern of glycolytic genes, the majority of the genes in the PPP and the incomplete TCA cycle were transcribed at similar levels under both conditions (Fig. 3). Therefore, the expression of most genes in glycolysis, PPP and the incomplete TCA cycle remained relatively stable expression levels no matter if glucose or lactate was utilized.

The reverse β-oxidation is known as a classical CoA-dependent chain elongation pathway to produce short- and medium-chain fatty acids such as butyrate and caproate in bacteria (41, 42). Remarkably, four genes (*hbd*, *crt*, *bcd*, and *cat*) of the reverse β-oxidation pathway and the nondecarboxylative condensation gene *thl* showed significantly increased expression with a minimum of 4-fold change under the lactate condition. Surprisingly, the transcripts of these five genes (*thl*, *hbd*, *crt*, *bcd*, and *cat*) accounted for 20% of the total transcripts under the lactate condition while they still accounted for 6% of the total transcripts under the glucose condition (Data set S1), indicating that the chain elongation using the reverse β-oxidation pathway played a crucial role in the survival of *C. lactatifermentans* regardless of glucose or lactate was utilized. Meanwhile, the reverse β-oxidation pathway is closely linked to membrane-bound energy conservation systems, e.g., the Rnf complex and the membrane-bound ATPase. As demonstrated in Clostridium kluyveri, a well-known caproate-producing bacterium, electron transport from reduced ferredoxin (Fd^2−^) to NAD^+^ is carried out by the Rnf complex and ion motive force (IMF) is generated, and then the IMF drives ATP synthesis via an F_0_F_1_-ATP synthase (43). The Rnf complex was also found in caproate-producing *C. lactatifermentans*, and the organization of the Rnf genes (GJQ69_04310 to GJQ69_04335 encoding *rnf*CDGEAB) is similar to that in Clostridium kluyveri (44). Furthermore, nine significantly upregulated genes (GJQ69_08575 to GJQ69_08615) with fold changes between 2.6 to 3.5 under lactate condition were annotated as V-type ATP synthase subunit D, B, A, F, C, E, K, I, and G, respectively (Fig. 3). Therefore, a complete V-type ATP synthase is present in *C. lactatifermentans* whereas F-type ATP synthase-encoding genes were not found, indicating that V-type ATP synthase rather than the F-type ATP synthase is responsible for the ATP production in *C. lactatifermentans*.

**FIG 4:**
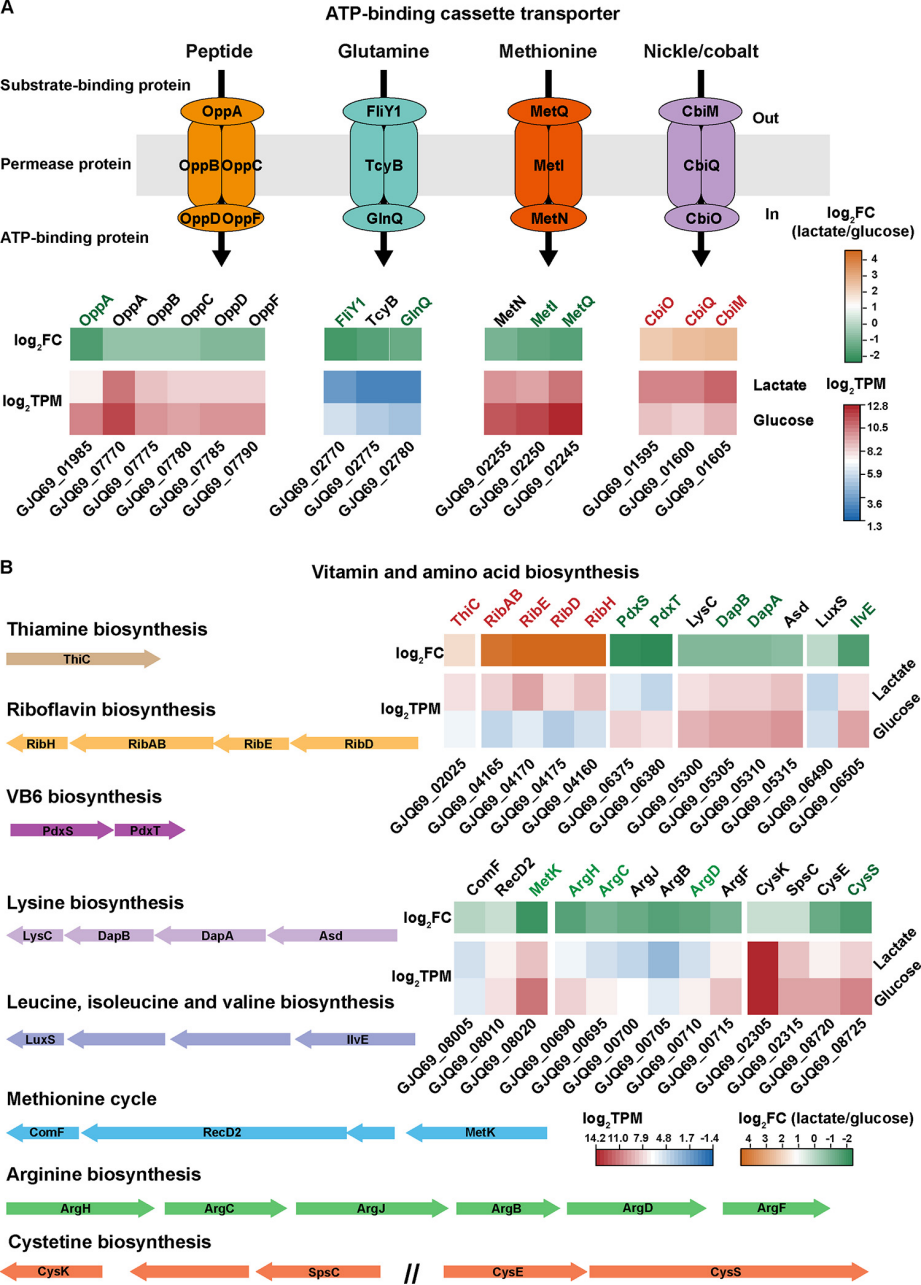
Gene expression patterns of (A) partial ABC transporters, and (B) amino acid metabolism and vitamin synthesis in *C. lactatifermentans*. The significantly upregulated genes under lactate conditions are shown in red, the significantly downregulated genes under lactate conditions are shown in green, and nonsignificantly changed genes are shown in black. ThiC, phosphomethylpyrimidine synthase; RibH, 6,7-dimethyl-8-ribityllumazine synthase; RibAB, 3,4-dihydroxy-2-butanone-4-phosphate synthase; RibE, riboflavin synthase; RibD, diaminohydroxyphosphoribosylaminopyrimidine deaminase; PdxS, pyridoxal 5′-phosphate synthase lyase subunit; PdxT, pyridoxal 5′-phosphate synthase glutaminase subunit; LysC, aspartate kinase; DapB, 4-hydroxy-tetrahydrodipicolinate reductase; DapA, 4-hydroxy-tetrahydrodipicolinate synthase; Asd, aspartate-semialdehyde dehydrogenase; LuxS, S-ribosylhomocysteine lyase; IlvE, branched-chain amino acid aminotransferase; ComF, competence protein F; RecD2, ATP-dependent RecD-like DNA helicase; MetK, methionine adenosyltransferase; ArgH, argininosuccinate synthase; ArgC, N-acetyl-gamma-glutamyl-phosphate reductase; ArgJ, glutamate N-acetyltransferase; ArgB, acetylglutamate kinase; ArgD, acetylornithine/succinyldiaminopimelate aminotransferase; ArgF, ornithine carbamoyltransferase; CysK, cysteine synthase; SpsC, aminotransferase class I/II-fold pyridoxal phosphate-dependent enzyme; CysE, serine acetyltransferase; CysS, cysteine-tRNA ligase.

For *C. lactatifermentans*, much more biomass was accumulated when glucose was used as carbon source than that of lactate (Fig. 1A). The precursors of biomass production, including amino acids, vitamins, and nucleotides, were derived from carbon metabolism upstream of acetyl-CoA synthesis. For *C. lactatifermentans*, some of these nutrients also need to be acquired from the surrounding environment because this small-genome bacterium is deficient in *de novo* synthesis of fundamental building blocks of the cell, e.g., 12 amino acids, biotin, folate, and nicotinate (19). Despite these deficiencies, when glucose was utilized as carbon source, the expression levels of genes responsible for peptide and amino acid transport were significantly upregulated. The upregulated genes include *metI* and *metQ* which encode methionine transporter proteins, gene *oppA* which encodes the peptide transporter protein, and genes *fliy1* and *glnQ* which encode glutamine transporter proteins (Fig. 4). Furthermore, the genes involved in the amino acid biosynthesis and metabolism pathways were also transcribed at significantly higher levels under the glucose condition (Fig. 4). For instance, gene *ilvE* (4.2-fold change) is involved in the last synthesis step of leucine, isoleucine, and valine biosynthesis, and genes *argH* (3.5-fold change), *argC* (2.5-fold change), and *argD* (3.2-fold change) are involved in arginine biosynthesis (Fig. 4). Besides, gene *metK* (GJQ69_008020) catalyzing the first step of the methionine cycle to produce S-adenosylmethionine (SAM) from methionine and ATP showed a 4.9-fold increased expression when glucose was utilized (Fig. 4). The methionine cycle provides methyl groups for many methylation reactions and plays an essential role in cell growth (45). In addition, *cysK* (GJQ69_02305), the gene involved in the last step of cysteine synthesis, was the seventh-highest expressed gene with a TPM of more than 15,000 under both conditions (Data set S1), indicating that this gene may play an important physiological function in strictly anaerobic *C. lactatifermentans*. What’s more, vitamins and their derivatives are important cofactors for many enzymes. The two subunits of pyridoxal 5′-phosphate synthase-encoding genes *pdxS* (5.2-fold change) and *pdxT* (6.3-fold change) involved in vitamin B6 biosynthesis, showed significantly increased expression under the glucose condition (Fig. 4). Considering the modified Clostridium growth medium (mCGM) containing 10 g · L^−1^ tryptone and 10 g · L^−1^ yeast extract was rich in amino acids and vitamins, we wondered how much active biosynthesis and metabolism of amino acids, peptides, and vitamins would contribute to biomass accumulation of *C. lactatifermentans*. *C. lactatifermentans* was cultured in the same medium without glucose and lactate. The maximum cell density (OD_600_) was 0.8 in the absence of glucose or lactate (Fig. S3), which was much lower than that when glucose was used as carbon source (Fig. 1A). Therefore, glucose was essential to the accumulation of biomass besides the sufficient supplies of amino acids and vitamins.

10.1128/msystems.00534-22.6 FIG S3 The cell growth and metabolite production of *C. lactatifermentans* in mCGM without glucose and lactate. Acetate and butyrate were supplemented in mCGM as electron receptors. Download FIG S3, EPS file, 0.8 MB. Copyright © 2022 Wang et al. 2022 Wang et al. https://creativecommons.org/licenses/by/4.0/ This content is distributed under the terms of the Creative Commons Attribution 4.0 International license.

On the contrary, pyruvate-ferredoxin oxidoreductase (Pfor)-encoding gene GJQ69_01365, responsible for acetyl-CoA generation from pyruvate, was the highest expressed gene (TPM of 47,829 in glucose and TPM of 79,387 in lactate) with a 2.6-fold increased expression under the lactate condition (Data set S1). Pyruvate-ferredoxin oxidoreductase requires thiamine diphosphate (TPP) as a cofactor. *ThiC* (GJQ69_02025), a gene involved in thiamine diphosphate biosynthesis, showed a 3.3-fold increased expression under the lactate condition (Fig. 4). The upregulation of *thiC* was likely to support the activity of pyruvate-ferredoxin oxidoreductase. Furthermore, several genes involved in riboflavin biosynthesis were upregulated under the lactate condition (Fig. 4), suggesting that riboflavin might be closely related to lactate utilization and caproate production rather than biomass production. More specifically, the significantly upregulated genes involved in riboflavin biosynthesis under lactate condition were: *ribH* with an 18.9-fold change, *ribBA* with a 24.2-fold change, *ribE* with a 23.9-fold change, and *ribD* with a 24.5-fold change. Riboflavin is the precursor for the synthesis of flavin adenine dinucleotide (FAD) which is required for FAD-dependent enzymes, e.g., lactate dehydrogenase (GJQ69_08645), butyryl-CoA dehydrogenase (GJQ69_08630), and electron transfer flavoproteins (GJQ69_08635 and GJQ69_08640). As indicated in Fig. 3, these FAD-dependent enzymes involved in lactate utilization and caproate production were all significantly upregulated under the lactate condition. Therefore, the active synthesis of riboflavin may mainly contribute to the above-mentioned FAD-containing holoenzyme assembly. Similarly, the upregulation of cobalt/nickel ABC transport (Fig. 4) was also related to lactate utilization considering a 16.3-fold increased expression of nickel-dependent lactate racemase gene *larA* under the lactate condition (Data set S1).

### Bioenergetics of glucose and lactate fermentation.

The reverse β-oxidation pathway is responsible for the redox balance of cells because the reducing power (NADH or NADPH) generated from reducing substrates such as ethanol, lactate, and glucose, are mainly consumed via reverse β-oxidation (46, 47). Acetate, one of the direct precursors of acetyl-CoA, provides a two-carbon unit into reverse β-oxidation accompanied by NADH (NADPH) consumption. Therefore, NADH (NADPH) availability may largely depend on the ratio of reducing substrate to electron acceptor such as acetate. It has been reported that the initial ratio of reducing substrate to electron acceptors (C2-C4 fatty acids) regulates the selectivity for short- and medium-chain fatty acids in chain elongation bacteria such as Clostridium kluyveri as well as chain elongation microbiota (4, 48–51). We speculated that the selectivity for medium-chain fatty acids might be closely linked to NADH availability. To explore the effects of reducing power availability on caproate production in *C. lactatifermentans*, the varying ratios of reducing substrate to acetate with the same glucose equivalent were set up.

**FIG 5:**
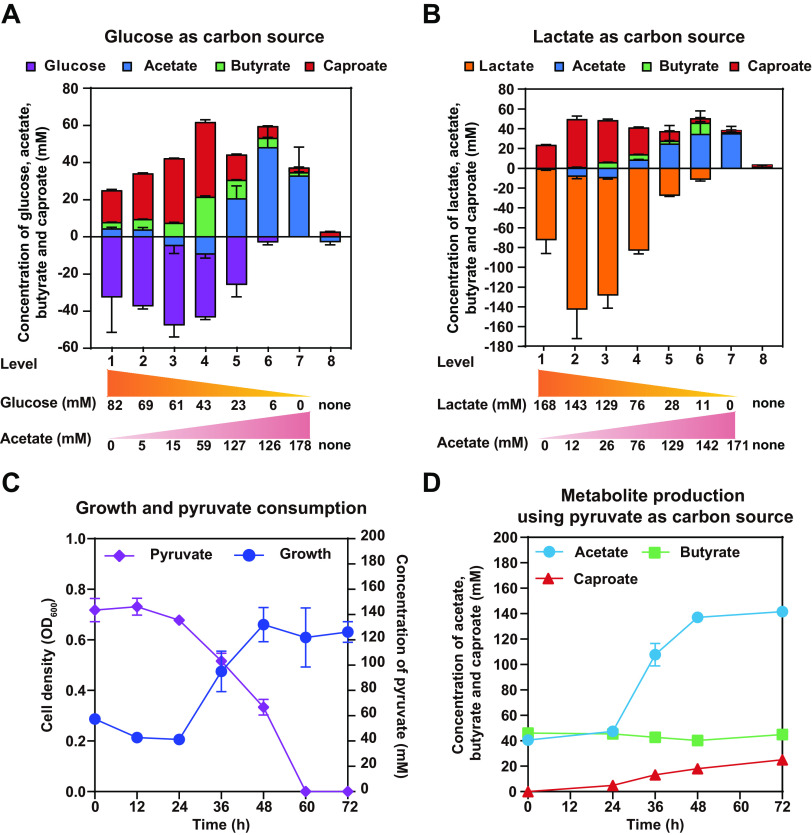
The types of reducing substrate and ratio of reducing substrate to acetate influenced the fermentation end products in *C. lactatifermentans*. (A) The different molar ratios of glucose to acetate influenced the ratio of fermentation end products. (B) The different molar ratios of lactate to acetate influenced the ratio of fermentation end products. (C) Cell growth and pyruvate consumption. (D) Acetate was the main fermentation end product when using pyruvate as a reducing substrate. Acetate and butyrate were supplemented as electron acceptors, and net accumulation of acetate and butyrate were demonstrated. Level 8 was indicated as a control group without acetate, glucose, and lactate.

**FIG 6:**
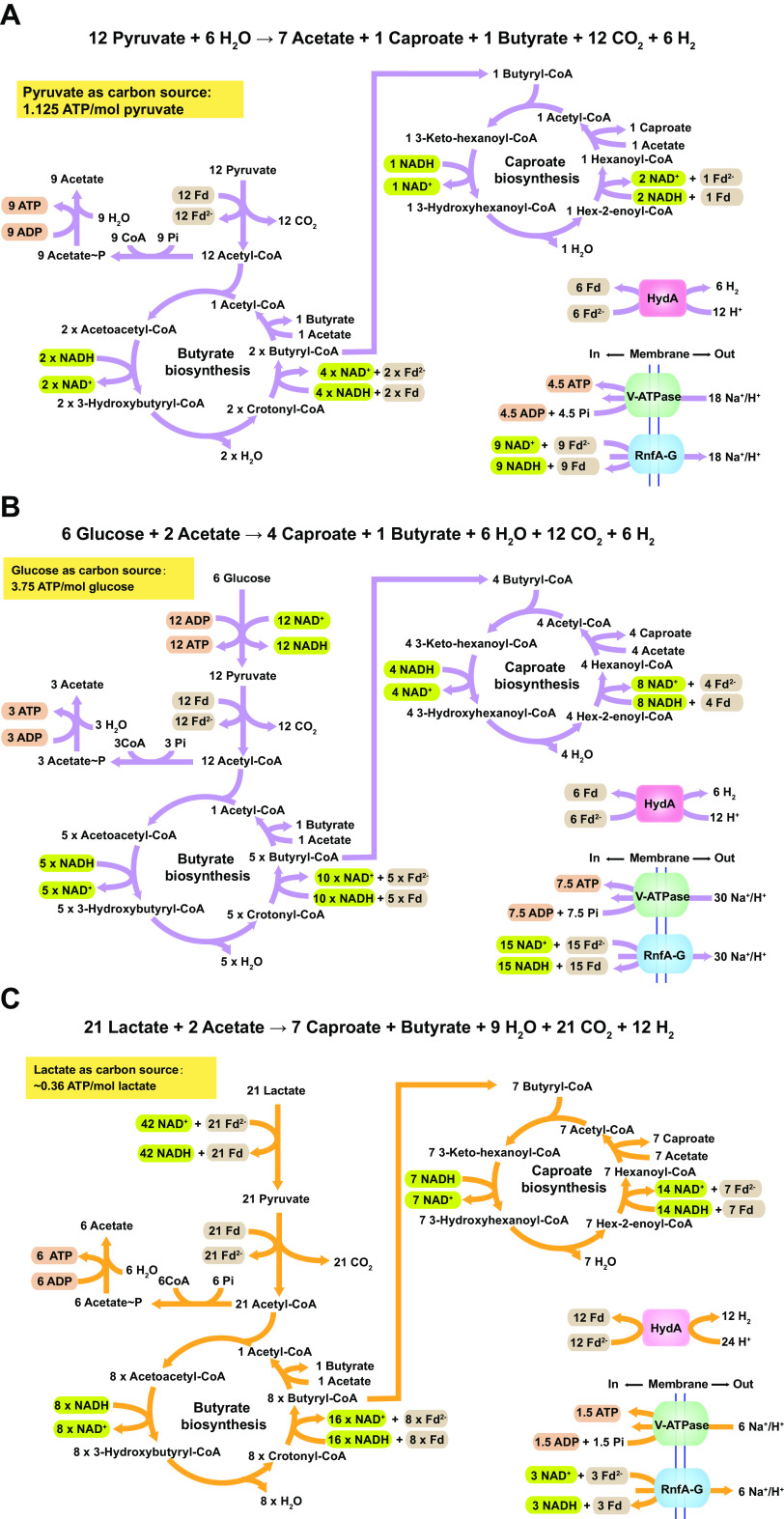
The caproate production from (A) pyruvate-based chain elongation, (B) glucose-based chain elongation, and (C) lactate-based chain elongation by *C. lactatifermentans*.

When acetate was not additionally supplemented into the medium (level 1), only a small amount of glucose or lactate was utilized (Fig. 5A and B), indicating that the reverse β-oxidation pathway was not fully engaged to drive the glucose and lactate utilization. Nevertheless, with the increase of acetate (level 2 to 4, with the ratio of reducing substrate to acetate less than 15), glucose and lactate utilization efficiencies were both gradually increased (Fig. 5A and B), which may be attributed to the sufficient supply of acetyl-CoA required for reverse β-oxidation. As indicated in Fig. 5A and B, the ratio of caproate to butyrate was decreased along with the increase of acetate from level 1 to 7. In other words, the higher concentration of glucose or lactate promoted higher specificity for caproate production, indicating that the selectivity for medium-chain fatty acids can be elevated when a higher ratio of reducing substrate to acetate was used. Interestingly, with the decrease of reducing substrate (level 5 to 7), considerable acetate was produced as the main fermentation product, but only small amounts of caproate and butyrate were produced (Fig. 5A and B). The low ratio of reducing substrate to acetate indicated low reducing power (NADH and NADPH) supply. To examine the importance of the glycolysis and lactate oxidation pathway on reducing power generation and fermentation end product synthesis, pyruvate, the product of the glycolysis pathway and the oxidated intermediate of lactate, was used as the reducing substrate. *C. lactatifermentans* showed weak growth when using pyruvate as reducing substrate (Fig. 5C). The fermentation using pyruvate as reducing substrate revealed that acetate rather than caproate was the main product though the electron acceptors (acetate and butyrate) were sufficient in the medium (Fig. 5D). Considering that the NADH provided by one mole of pyruvate is only one-quarter of the NADH provided by the same mole of glucose, or one-half of the NADH provided by the same mole of lactate. It is quite possible that when reducing power was insufficient, more acetyl-CoA had to be converted into acetate via the acetate kinase-phosphate acetyltransferase (Ack-Pta) pathway rather than caproate via the reverse β-oxidation pathway (Fig. 6A). Therefore, when reducing substrate (e.g., glucose or lactate) was insufficient, the main fermentation end product would be acetate, which can be produced from pyruvate and several amino acids.

The above fermentation tests suggested that reducing power (NADH or NADPH) availability affected the ratio of end products during fermentation. The higher NADH (NADPH) availability might result in higher selectivity for caproate rather than acetate or butyrate. It should be noticed that the selectivity for caproate was higher when lactate was used as a reducing substrate rather than glucose under the same molar ratio of glucose to acetate and lactate to acetate (Fig. 5A and B), implying that NADH (NADPH) availability could be higher under lactate conditions than glucose conditions. Furthermore, the reverse β-oxidation pathway was more active under the lactate condition (Fig. 3). We then questioned how the reducing power availability affected the expression of caproate production machinery such as thiolase and the reverse β-oxidation pathway.

It has been well known that Rex, a global transcriptional regulator, usually responds to cellular NADH/NAD^+^ ratio, playing a key role in regulating cellular carbon and energy metabolism in bacteria belonging to the phyla Firmicutes (52–55). Hence, we questioned whether a Rex in *C. lactatifermentans* was involved in regulating caproate production. Then a putative Rex regulator encoded by gene GJQ69_04720 was identified by using the *rex* gene from Clostridium kluyveri (52) to search all the 1,836 coding DNA sequences (CDS) in the genome of *C. lactatifermentans* by protein BLAST. Unlike the tight linkage of *rex* with reverse β-oxidation genes in other species such as Clostridium kluyveri and Clostridium acetobutylicum (52, 53), *rex* in the genome of *C. lactatifermentans* is far from the gene cluster of reverse β-oxidation. Specifically, *rex* of *C. lactatifermentans* is located in the operon containing two TCA cycle genes, i.e., *acn* and *icd*, which encode aconitate hydratase and isocitrate dehydrogenase, respectively (Fig. S4A). The average transcriptional levels (TPM) of *rex* were 955 and 628 under glucose and lactate conditions, respectively, while its transcription (fold change = 1.0, *P* value = 0.99) showed no significant difference between these two different conditions, suggesting that neither glucose nor lactate changed the transcription level of *rex* in *C. lactatifermentans*. Subsequently, a typical Rex binding motif “TTGTTAANNNNTTAACAA” in Clostridiaceae (52) was used as the seed motif to predict potential Rex-binding sites in *C. lactatifermentans* by FIMO in MEME Suite 5.1.1 (56). A total of 27 Rex-binding sites were detected within intergenic regions. The consensus motif of these Rex-binding sites was visualized in Fig. S4B by using Weblogo (57). *In silico* analysis predicted Rex to bind to the 5′-untranslated region (UTR) of genes mainly involved in carbon and energy metabolism in *C. lactatifermentans* (Table 1). Specifically, the genes with potential Rex-binding sites were mainly involved in lactate utilization, the reverse β-oxidation, the TCA cycle, and the Rnf complex (Table 1). The possible control of Rex on the reverse β-oxidation operon *thl*-*crt*-*hbd* and gene *cat* is illustrated in Fig. S4C. In addition, a putative Rex-binding site located in the 5′-UTR of the *ldh-etfB-etfA-bcd* operon (GIQ69_008630 to GIQ69_008650) was also found (Table 1). *Ldh* and *bcd* encode lactate dehydrogenase and butyryl-CoA dehydrogenase, respectively, implying that Rex might simultaneously control lactate utilization and caproate production. Considering that Rex is a transcriptional repressor, and its binding activity is usually repressed by elevated NADH level (52), the expression of these reverse β-oxidation genes in *C. lactatifermentans* might be controlled by Rex, thus contributing to maintaining the cellular redox balance.

**TABLE 1:** The genes potentially regulated by Rex in *C. lactatifermentans*

Operon	The first gene of the operon				Operon function	Predicted Rex-binding position	Predicted Rex-binding site	Score
	Locus tag	TPM (glucose)	TPM (lactate)	log_2_FC (lactate/glucose)				
*acn-*GJQ69_04730-*icd-rex*	GJQ69_04735	1,716	1,400	0.31	Aconitate hydratase	−243	TTGTGAATGGATTGACAA	14.82
*thl-crt-hbd*	GJQ69_04190	18,250	73,350	2.79	Caproate production	−151	TTGCTAAAACTTTAACAA	19.79
*cat*	GJQ69_04210	3,153	7,806	2.02	Butyryl-CoA:acetate CoA-transferase	−77	TAGTTACTTTATTAACAA	14.32
*ldh-etfB-etfA-bcd*	GJQ69_08645	6,294	35,189	3.18	Lactate utilization	−81	TTTTTTAAGAATTAACAC	9.07
*rnfCDGEAB*	GJQ69_04335	1,032	367	−1.04	Electron transport complex	−101	TTATTCAATCTTTAACAA	14.29
*rpe*	GJQ69_09515	118	624	3.05	Ribulose-phosphate 3-epimerase	−123	TTTTTAACCGCTTAATCA	9.55
*hydA*	GJQ69_02965	1,136	1,893	1.55	[FeFe]-hydrogenase	−117	TTGTTATAATAAAAACAA	9.09
	GJQ69_09080	122	81	0.07	SDR family NAD(P)-dependent oxidoreductase	−148	TTGTAAAACAAAAAACAA	9.35

10.1128/msystems.00534-22.7 FIG S4 The redox-sensing transcriptional repressor Rex might regulate the genes involved in the TCA cycle and reverse β-oxidation pathway. (A) The organization of the gene cluster containing *rex* gene. (B) Visualization of the consensus sequence of putative Rex-binding site in *C. lactatifermentans*. The consensus motif was generated from all of the predicted Rex-binding sites. (C) The putative Rex-binding site, and the -10 and -35 promoter sequences preceding genes *thl* and *cat* were indicated. Acn, aconitate hydratase; Icd, isocitrate dehydrogenase; Thl, thiolase; Crt, enoyl-CoA hydratase; Hbd, 3-hydroxybutyryl-CoA dehydrogenase; Cat, butyryl-CoA: acetate CoA transferase. Download FIG S4, EPS file, 1.3 MB. Copyright © 2022 Wang et al. 2022 Wang et al. https://creativecommons.org/licenses/by/4.0/ This content is distributed under the terms of the Creative Commons Attribution 4.0 International license.

The typical Rex binding motif “TTGTTAANNNNTTAACAA” in Clostridiaceae (52) was also used to predict the potential Rex-binding sites in the genome of *C. amylolyticum*, the closest relative of *C. lactatifermentans*. As indicated in Table S1, similar to *C. lactatifermentans*, the genes with potential Rex-binding sites were also involved in the reverse β-oxidation, the TCA cycle, and the Rnf complex, suggesting that the regulation of Rex on these metabolic pathways may be conserved in the genus *Caproicibacterium* though *C. lactatifermentans* is a lactate-utilizing bacterium whereas *C. amylolyticum* is a lactate-producing bacterium (37, 58).

10.1128/msystems.00534-22.1 TABLE S1 The genes potentially regulated by Rex in *C. amylolyticum*. Download Table S1, XLSX file, 0.02 MB. Copyright © 2022 Wang et al. 2022 Wang et al. https://creativecommons.org/licenses/by/4.0/ This content is distributed under the terms of the Creative Commons Attribution 4.0 International license.

To quantitatively compare the energy production when using glucose or lactate as reducing substrate, the stoichiometric equations (equation 1 and equation 2) of caproate fermentation were built based on the actual substrate consumption and metabolite production under a fixed reducing substrate to acetate molar ratio of 5:1 (level 3). The thermodynamics were calculated by using eQuilibrator (59). ΔG^0^′ values for equations 1 and 2 were normalized to per mole of glucose and per mole of lactate, respectively.

(1) $${\text{6CH}}_{\text{2}}\text{OH}{\left(\text{CHOH}\right)}_{\text{4}}\text{CHO}+{\text{ 2CH}}_{\text{3}}{\text{COO}}^{-}\to {\text{ 4CH}}_{\text{3}}{\left({\text{CH}}_{\text{2}}\right)}_{\text{4}}{\text{COO}}^{-}+{\text{CH}}_{\text{3}}{\left({\text{CH}}_{\text{2}}\right)}_{\text{2}}{\text{COO}}^{-}+{\text{3 H}}^{+}+{\text{6 H}}_{\text{2}}\text{O }+{\text{ 12 CO}}_{\text{2}}+{\text{ 6 H}}_{\text{2}}$$

$$\Delta {\text{G}}^{0\prime }\text{per mol glucose}=-\text{254}.\text{2 }\pm \text{ 13}.\text{2kJ}\cdot {\text{mol}}^{-\text{1}}$$

(2) $${\text{21CH}}_{\text{3}}\text{CH}\left(\text{OH}\right){\text{COO}}^{-}+{\text{ 2CH}}_{\text{3}}{\text{COO}}^{-}+{\text{ 15 H}}^{+}\to {\text{ 7CH}}_{\text{3}}{\left({\text{CH}}_{\text{2}}\right)}_{\text{4}}{\text{COO}}^{-}+{\text{CH}}_{\text{3}}{\left({\text{CH}}_{\text{2}}\right)}_{\text{2}}{\text{COO}}^{-}+{\text{ 9 H}}_{\text{2}}\text{O }+{\text{ 21 CO}}_{\text{2}}+{\text{ 12 H}}_{\text{2}}$$

$$\Delta {\text{G}}^{0\prime }\text{per mol lactate }=\;-\text{24}.\text{6 }\pm \text{ 7}.0\text{ kJ}\cdot {\text{mol}}^{-\text{1}}$$

Furthermore, the metabolic pathways of caproate production from glucose or lactate were reconstructed while considering biochemical stoichiometry, redox balance, and ATP synthesis (Fig. 6B and C). Based on the H^+^/ATP ratio of the V-type ATPase determined from Thermus thermophilus (60), the H^+^/ATP ratio of the V- type ATPase in *C. lactatifermentans* was defined as 4 in this study. According to the reconstructed metabolic pathways, 3.75 mol of ATP and around 0.72 mol of ATP can be synthesized from one mole of glucose and two moles of lactate, respectively. Therefore, it can be calculated that 68 kJ free energy was required for synthesis of one mole of ATP when glucose or lactate was used as a reducing substrate. The Gibbs free energy required for the generation of per mole ATP in *C. lactatifermentans* were comparable to the value (72 kJ) that has been reported for Clostridium kluyveri (10). Furthermore, the ATP yield from one mole of glucose was around five times as much as that from two moles of lactate, suggesting that lactate was not an ideal carbon source like glucose if considering the ATP yield. Moreover, glucose can provide more intermediates from the glycolysis and the PPP pathway than lactate to support biomass synthesis. Together, from the perspectives of energy and metabolite intermediate supply, glucose is a better carbon source for the growth of *C. lactatifermentans* compared to lactate.

### Glucose negatively modulates lactate utilization through a transcriptional repressor LldR.

Based on the above bioenergetic analysis, *C. lactatifermentans* harvested much less ATP by using lactate rather than glucose (Fig. 6B and C), indicating that the significant upregulation of the lactate utilization genes and the reverse β-oxidation genes is essential for bacterium survival on lactate. The three lactate utilization genes, *lldP*, *larA*, and *ldh* with TPM of 11,321; 16,133; and 35189, respectively, accounted for around 6.2% of the total transcripts under lactate conditions, whereas these lactate utilization genes showed at least a 5.6-fold decrease under glucose conditions (Data set S1). We were curious whether the expressions of these lactate utilization genes can be regulated, and if they can be still highly expressed when glucose and lactate coexist.

**FIG 7:**
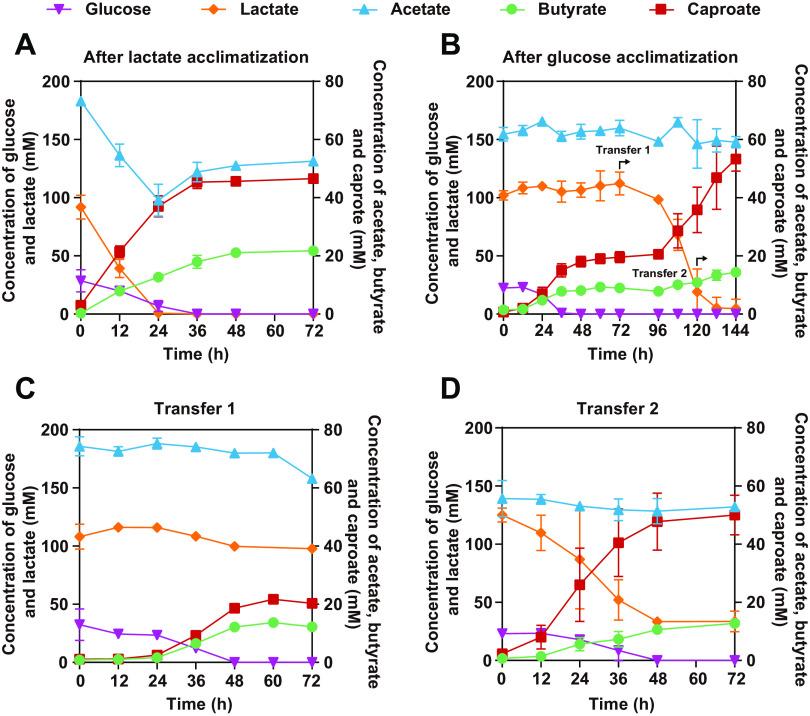
Glucose availability reduced lactate utilization of *C. lactatifermentans*. (A) Glucose and lactate were coutilized after lactate acclimatization in a lactate-rich *Baijiu* fermentation system. (B) Glucose was preferentially utilized when glucose and lactate coexisted in the medium after glucose acclimatization. (C) The lactate utilization cannot be restored by transferring the primary culture at 72 h. (D) Recovery of glucose and lactate coutilization capability by transferring the primary culture at 120 h.

As demonstrated in our prior study (19) and Fig. 7A, after long-term lactate acclimatization, lactate can be simultaneously utilized with glucose by *C. lactatifermentans*, indicating that lactate utilization genes were actively expressed when glucose and lactate coexisted. However, it was still unknown whether the expression of the lactate utilization genes could be affected if the cells were subjected to glucose acclimatization. To determine the effect of glucose acclimatization on lactate utilization, successive transfers were performed in glucose-containing mCGM for around 1 month. After the acclimatization on glucose medium, *C. lactatifermentans* preferentially utilized glucose though lactate and glucose were both present in the medium (Fig. 7B). Interestingly, lactate utilization cannot be immediately restored after glucose exhaustion, but can be restored at 36 h post glucose exhaustion, and thus a second caproate production phase was observed (Fig. 7B). This finding indicated that glucose acclimatization impaired lactate utilization for *C. lactatifermentans*. Moreover, to determine whether this inhibition on lactate utilization is temporary or permanent, we transferred the primary culture when lactate utilization had not been restored (72 h) and had been restored (120 h). When the inoculated seed was transferred at 72 h, the inhibition on lactate utilization remained in the presence of glucose (Fig. 7C). On the contrary, when the inoculated seed was transferred at 120 h, the time point of lactate utilization recovery, the simultaneous utilization of lactate and glucose was observed (Fig. 7D), indicating that lactate utilization cannot be inhibited anymore once lactate utilization was restored even if glucose was available. Therefore, we speculated that lactate utilization was temporarily inhibited only when glucose was present for a long time and lactate was absent. It is intriguing how glucose temporarily inhibited lactate utilization.

**FIG 8:**
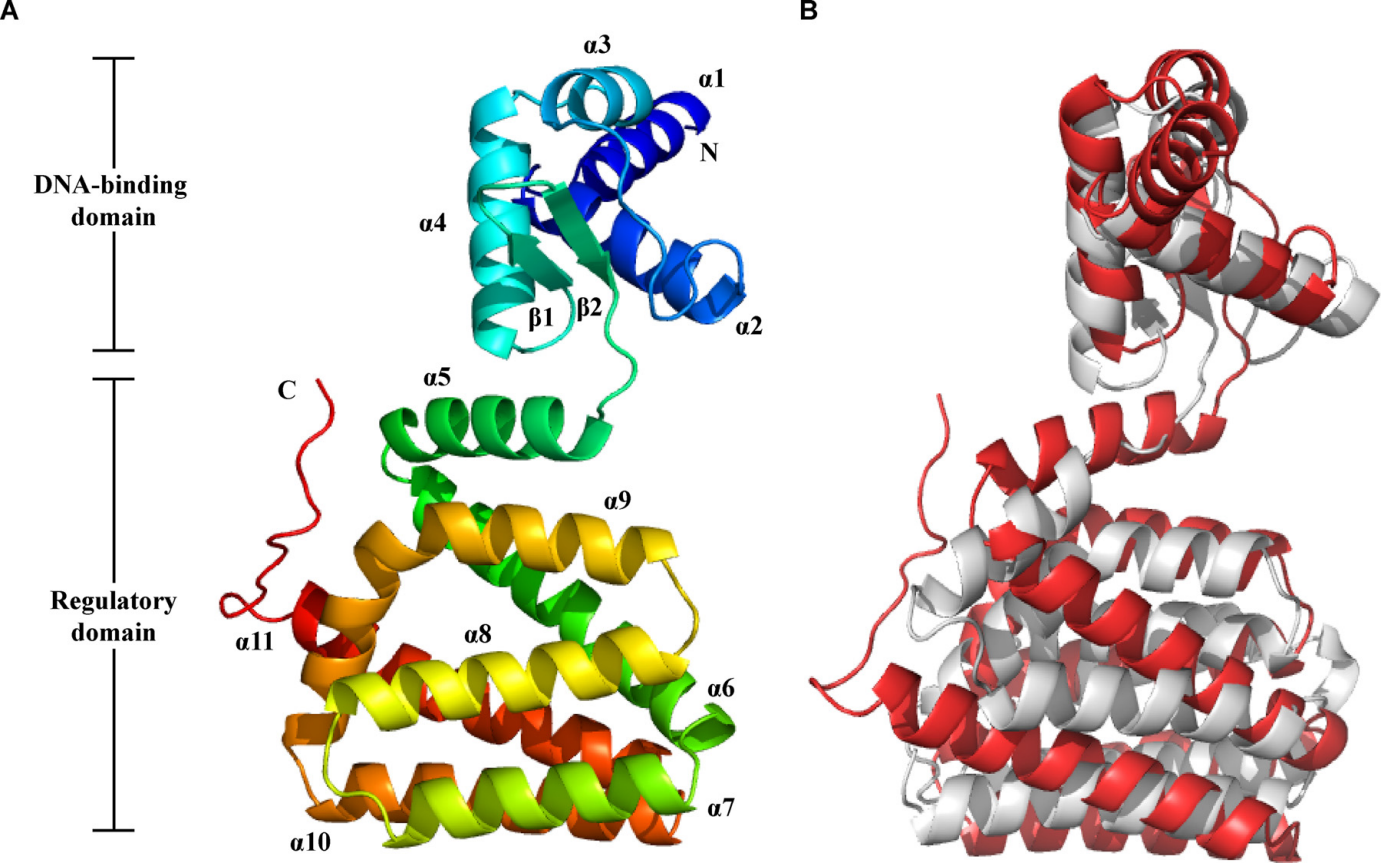
A protein belonging to the GntR family was predicted as the lactate utilization repressor LldR in *C. lactatifermentans*. (A) The predicted tertiary structure of putative LldR protein from *C. lactatifermentans*. (B) Alignment of LldR from *C. lactatifermentans* (indicated in red) with LldR from C. glutamicum (indicated in gray, Protein Data Bank code: 2DI3) by PyMol v2.5.2 (93).

Previous studies have reported that LldR, a transcriptional regulator belonging to the GntR family, acts as a transcriptional repressor for lactate utilization in several lactate-utilizing bacteria, such as Corynebacterium glutamicum (61), Acetobacterium woodii (22), Pseudomonas aeruginosa (62), Bacillus subtilis (63), and Escherichia coli (64). Among these bacteria, *A. woodii* and *C. lactatifermentans* both belong to the class Clostridia. The amino acid sequence of LldR in *A. woodii* DSM 1030 was chosen to search for the potential LldR in the genome of *C. lactatifermentans*. Among all the 1,836 proteins in the genome of *C. lactatifermentans*, the protein encoded by gene GJQ69_00910 had the highest similarity of 62.7% to the LldR protein from *A. woodii*. Hence, the protein encoded by gene GJQ69_00910 was considered the most likely candidate for LldR in *C. lactatifermentans*. Subsequently, the protein sequence similarities were examined between LldR from *C. lactatifermentans* and the LldR proteins from C. glutamicum ATCC 13032, *A. woodii* DSM 1030, P. aeruginosa XMG, E. coli MG1655, and B. subtilis RO-NN-1 (Fig. S5A). The similarities ranged from 46.7% to 62.7%, implying the cross-class conservation of the LldR protein. By using the Prabi server (https://npsa-prabi.ibcp.fr/cgi-bin/npsa_automat.pl?page=/NPSA/npsa_hth.html), a typical prokaryotic HTH DNA-binding domain was found at the N-terminal of LldR from *C. lactatifermentans*. Besides, the five conserved amino acid residues (Arg116, Asp164, His168, His217, and His239) involved in Zn^2+^ binding were found in the amino acid sequence of the LldR protein from *C. lactatifermentans* (Fig. S5B), indicating that the LldR protein from *C. lactatifermentans* has the common primary structural feature with the other LldR proteins (61). Subsequently, the tertiary structure of *C. lactatifermentans* LldR was predicted by the Robetta online tool (65) (Fig. 8A), and subjected to alignment with the classical structure of LldR from C. glutamicum (61). The overall structures of the two LldR proteins were quite similar though these two bacteria belong to different classes (Fig. 8B). The above primary and tertiary structure analysis suggested that the protein encoded by gene GJQ69_00910 was likely to be a lactate utilization repressor in *C. lactatifermentans*.

10.1128/msystems.00534-22.8 FIG S5 The protein sequence comparison of the potential lactate utilization repressor LldR in *C. lactatifermentans* with that of C. glutamicum, *A. woodii*, P. aeruginosa, E. coli, and B. subtilis. (A) The phylogeny and similarity of potential LldR in *C. lactatifermentans* with LldR from the other bacteria. (B) The protein sequence alignment of potential LldR in *C. lactatifermentans* with LldR from the other bacteria. The identical and similar amino acid residues with a threshold of 70% were highlighted by orange and grey shaded boxes, respectively. The blue underline indicated the predicted HTH DNA-binding domain of LldR in *C. lactatifermentans*. The amino acid residues involved in Zn^2+^ binding in LldR were indicated by red asterisks. Download FIG S5, EPS file, 2.2 MB. Copyright © 2022 Wang et al. 2022 Wang et al. https://creativecommons.org/licenses/by/4.0/ This content is distributed under the terms of the Creative Commons Attribution 4.0 International license.

**FIG 9:**
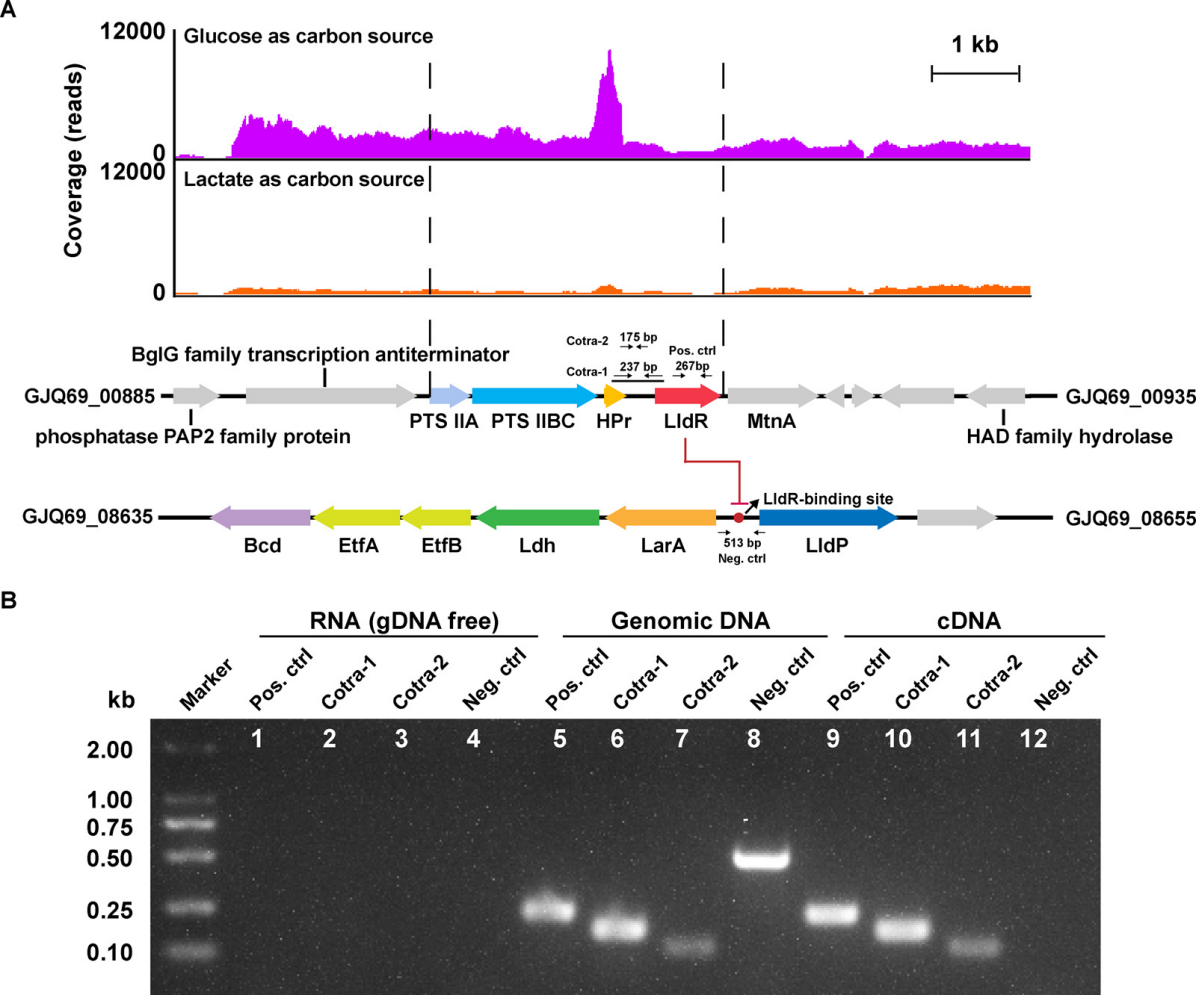
Glucose catabolism affected the transcription of the lactate utilization repressor, LldR. (A) The transcriptional patterns of PTS genes and the *lldR* gene in *C. lactatifermentans* under glucose and lactate conditions. (B) Verification of the cotranscription of *lldR* (GJQ69_00910) and the phosphoryl carrier protein (HPr)-encoding gene (GJQ69_00905) by using RT-PCR. Two sets of primer pairs (Cotra-1 and Cotra-2) were designed to amplify different parts of the intergenic region between *lldR* and the HPr-encoding gene. The coding region of *lldR* was used as the positive control, and the intergenic region between *larA* and *lldP* was used as the negative control. Genomic DNA was amplified to ensure PCR amplification worked. RNA with genomic DNA removal was also used as a negative control to ensure that genomic DNA had been completely depleted. Lanes 1, 5, and 9, positive control; lanes 2, 6, and 10, the intergenic region amplified by primer pair Cotra-1; lanes 3, 7 and 11, the intergenic region amplified by primer pair Cotra-2; and lanes 4, 8, and 12, negative control.

Different from that, the *lldR* gene is usually located adjacent to lactate utilization genes (22, 62, 63, 66), but the *lldR* gene (GJQ69_00910) in *C. lactatifermentans* is located downstream of a PTS operon which consists of genes encoding PTS enzyme II (GJQ69_00895, GJQ69_00900) and a phosphoryl carrier protein (HPr, GJQ69_00905) (Fig. 9A). Genes GJQ69_00895 and GJQ69_00900 encode PTS subunits IIA and IIBC, respectively, both of which showed more than 4-fold increased expression under glucose conditions (Data set S1). Besides, the HPr-encoding gene GJQ69_00905 showed an 8.1-fold increased expression under glucose conditions, while the other two HPr-encoding genes, GJQ69_00385 and GJQ69_02120, showed 1.1- and 1.9-fold increased expression under glucose conditions, respectively (Data set S1). As indicated in Fig. 9A, the significantly higher expression of the PTS operon (GJQ69_00895-GJQ69_00905) under glucose conditions rather than lactate conditions indicated that this PTS operon could be the main PTS responsible for glucose catabolism of *C. lactatifermentans*. What’s more, the *lldR* gene (GJQ69_00910) and the *hpr* gene (GJQ69_00905) were cotranscribed through a relatively long intergenic region (183 bp) between these two genes (Fig. 9). The cotranscription of the *hpr* gene and *lldR* genes enabled the expression of *lldR* to maintain a much higher expression level under glucose conditions than that under lactate conditions (Fig. S1E). The close linkage of the *lldR* gene with PTS genes in *C. lactatifermentans* may explain the finding of inhibition for lactate utilization by glucose acclimatization (Fig. 7B and C).

The consensus LldR-binding motif “TGGTNNNACCA” obtained from *A. woodii*, C. glutamicum, and P. aeruginosa (22, 61, 62) was used as the seed motif to predict potential LldR-binding sites in *C. lactatifermentans*. A potential LldR-binding site was located in the intergenic region between lactate racemase-encoding gene *larA* and lactate permease-encoding gene *lldP* (Fig. 9A). The *larA* gene and lactate dehydrogenase-encoding gene *ldh* were cotranscribed, and were divergently transcribed from *lldP* (Fig. S6). According to the RNA-seq analysis and real-time quantitative PCR, *ldh*, *larA*, and *lldP* showed the similar expression pattern that they were significantly downregulated under glucose conditions compared to lactate conditions (Fig. S1F, G and H). Therefore, the LldR protein was likely to negatively control the expressions of the three lactate utilization genes in *C. lactatifermentans*.

10.1128/msystems.00534-22.9 FIG S6 Identification of the cotranscription of lactate dehydrogenase gene *ldh* and lactate racemase gene *larA* in *C. lactatifermentans* by using RT-PCR. The primer pair Cotra-3 was designed to amplify the intergenic region between *ldh* and *larA*. The coding region of *ldh* was used as the positive control, and the intergenic region between *larA* and *lldP* was used as the negative control. Genomic DNA was amplified to ensure PCR amplification worked. RNA with genomic DNA removal was also used as a negative control to ensure that genomic DNA had been completely depleted. Lane 1, 4, and 7, the intergenic region between *ldh* and *larA*; Lane 2, 5, and 8, positive control; Lane 3, 6, and 9, negative control. Download FIG S6, TIF file, 2.2 MB. Copyright © 2022 Wang et al. 2022 Wang et al. https://creativecommons.org/licenses/by/4.0/ This content is distributed under the terms of the Creative Commons Attribution 4.0 International license.

## DISCUSSION

The increasing environmental concerns urge the replacement of petroleum-based feedstocks with biomass- and waste-derived feedstocks to produce high value-added chemicals in the field of industrial biotechnology (67–70). Medium-chain fatty acids (MCFAs) are one of the attractive products from these renewable feedstocks, such as lignocellulose and food wastes (71–73). Given the complex substrate composition in these renewable feedstocks, MCFA-producing bacteria frequently encounter mixtures of carbon sources. The recently published and validated *C. lactatifermentans* has a substantial industrial value due to its capability to produce caproate as the main fermentation metabolite utilizing mixed carbon sources such as lactate and glucose. Although it has been observed that the yield and productivity of caproate were closely related to the types of carbon sources in several caproate-producing bacteria (25, 26, 37), the molecular mechanisms of these selective phenomena have not been well understood. In this study, transcriptomic and bioenergetic studies were employed to understand the cellular responses of *C. lactatifermentans* to glucose and lactate. RNA-seq analysis revealed that the five genes (*thl*, *hbd*, *crt*, *bcd*, and *cat*) belonging to the reverse β-oxidation pathway showed significant upregulation when *C. lactatifermentans* was grown on lactate. Similarly, a previous study on Ruminococcaceae bacterium CPB6 (another strain of *C. lactatifermentans*) reported that the five reverse β-oxidation genes also showed significantly higher expression levels under glucose plus lactate conditions than sole glucose conditions (74). The reverse β-oxidation pathway was upregulated regardless of the presence of glucose, supporting the speculation that robust caproate production from lactate was triggered by increased intracellular NADH availability rather than decreased ATP level under lactate condition.

In contrast, compared to glucose, lactate is a less preferred carbon source for *C. lactatifermentans* because only limited biomass can be accumulated. Likewise, the growth defect culturing on lactate has also been reported in other bacteria, e.g., *A. woodii* (22) and *M. hexanoica* (25). Low cell density will not help to improve the titer and productivity when scaling-up caproate production by using lactate as a carbon source. The studies on Ruminococcaceae bacterium CPB6 and *M. hexanoica* have both demonstrated that hexose addition (glucose or fructose) can improve caproate production (25, 75) when lactate was utilized as the dominant carbon source. The biochemical mechanism underlying this phenomenon may be attributed to that hexose supplementation improves cell growth through providing more intermediates for biomass synthesis and elevating intracellular ATP level.

Lactate utilization has been well studied in several bacteria, such as *A. woodii*, C. glutamicum, B. subtilis, Shewanella oneidensis, and Pseudomonas putida (61, 76–79). For *C. lactatifermentans*, lactate oxidation is catalyzed by an electron confurcating lactate dehydrogenase/electron transferring flavoprotein complex (Ldh/EtfAB). This lactate utilization mode was first reported in *A. woodii* and was gradually found in other chain-elongation bacteria, such as Clostridium butyricum, Ruminococcaceae BL-4, Ruminococcaceae BL-6, *Anaerobutyricum soehngenii*, and an uncultured elongation bacterium “*Candidatus* Pseudoramibacter fermentans” (21, 27, 80, 81). EtfAB, an important electron carrier in many anaerobes, usually functions as an electron bifurcating complex during the reduction of enoyl-CoA, such as crotonyl-CoA and hex-2-enoyl-CoA (47, 82). For lactate-utilizing Clostridium butyricum, two sets of EtfAB complexes are present in its genome, with one specifically involved in lactate utilization and the other one specifically involved in enoyl-CoA reduction (21). Interestingly, only one set of EtfAB complex was found in *C. lactatifermentans*. Thus, the only EtfAB complex in *C. lactatifermentans* is involved in lactate oxidation as well as enoyl-CoA reduction.

Regarding the regulation of lactate utilization, for *A. woodii*, C. glutamicum, and B. subtilis, lactate utilization repressor LldR negatively regulates the transcription of lactate utilization genes in the absence of lactate (22, 76, 83). Nevertheless, the regulation of lactate metabolism in caproate-producing *C. lactatifermentans* remains poorly understood. In our previous study for *C. lactatifermentans*, glucose and lactate can be coutilized (19). However, the unexpected sequential utilization of glucose and lactate occurred after glucose acclimation, suggesting that lactate utilization was also negatively regulated by glucose in *C. lactatifermentans*. To clarify the regulation of glucose on lactate utilization, a putative lactate utilization repressor LldR was identified in *C. lactatifermentans*. Intriguingly, the *lldR* gene in *C. lactatifermentans* is not located adjacent to the lactate utilization operon like other reported *lldR* genes (22, 62, 63), but is included in a sugar-specific PTS operon, indicating that the inhibition of lactate utilization could be enhanced by glucose catabolism. Therefore, in *C. lactatifermentans*, not only lactate but also glucose regulates lactate utilization. Considering the extremely high expression levels of the three lactate utilization genes (*ldh*, *larA*, and *lldP*) under lactate conditions, the strict linkage of the PTS genes and the *lldR* gene can save energy and resources for various physiological and metabolic activities with preferred glucose as the sole carbon source in the medium.

Surprisingly, the species closely related to *C. lactatifermentans* were also distributed in other lactate-based chain elongation systems beyond *Baijiu* fermentation system, such as granular fermentation and a food waste anaerobic digestion (73, 84), addressing the substantial value of this caproate-producing bacterium being applied in diverse chain elongation systems. Nevertheless, it is still challenging to obtain robust caproate production by enrichment and maintenance of efficient chain elongation bacteria. Previously, the maximum caproate concentration of 33.7 g · L^−1^ was achieved by a lactate-based chain elongation microbiota dominated by strain CPB6 (another strain of *C. lactatifermentans*) in a 780-day fermentation (85). Understanding the physiological and metabolic mechanisms of the key caproate-producing bacterium will be greatly helpful to determine operation parameters to shorten the enrichment period of the caproate-producing microbiota and to maintain stable caproate production.

In conclusion, this study revealed the biochemical and genetic mechanisms underlying the robust growth of *C. lactatifermentans* on glucose and active caproate production on lactate. A novel regulation mechanism on lactate utilization by the lactate utilization repressor LldR in a glucose-dependent manner was revealed, suggesting an elaborate energy-saving mechanism in this small genome-sized bacterium. The in-depth understanding of carbon and energy metabolism in *C. lactatifermentans* lays the groundwork for future genetic engineering to optimize microbial cell factory for MCFA production.

## MATERIALS AND METHODS

### Medium.

The modified Clostridium growth medium (mCGM) contained (per L): tryptone 10 g, yeast extract 10 g, (NH_4_)_2_SO_4_ 2 g, sodium acetate 5 g, NaH_2_PO_4_ 1 g, K_2_HPO_4_ 0.5 g, MgSO_4_·7H_2_O 0.1 g, FeSO_4_·7H_2_O 0.015 g, MnSO_4_·H_2_O 0.01 g, CaCl_2_ 0.01 g, CoCl_2_ 0.002 g, and ZnSO_4_ 0.002 g. The medium preparation was the same as previously described (19).

### RNA extraction and transcriptome sequencing.

*Caproicibacterium lactatifermentans* was precultured in mCGM supplemented with glucose or lactate as the carbon source and then the pregrown culture was incubated into fresh mCGM for RNA preparation. When the cells were grown to the mid-exponential phase, three biological samples were collected by centrifugation at 10,000 × *g* for 8 min at 4°C, and then were ground into powder under the protection of liquid nitrogen, followed by resuspending in RNAiso Plus reagent (TaKaRa, Dalian, China). The total RNA was isolated according to the manufacturer’s instructions of the RNAiso Plus reagent. The quality and purity of extracted RNA samples were assessed by using NanoDrop spectrophotometry (Thermo Scientific, Waltham, MA) and Agilent 2100 bioanalyzer (Agilent Technologies, Palo Alto, CA), respectively. rRNA was subsequently depleted from total RNA using the Ribo-off rRNA depletion kit for bacteria (Vazyme, Nanjing, China). cDNA libraries were prepared with NEBNext Ultra directional RNA library prep kit for Illumina (New England BioLabs, Ipswich, MA). Then 150-bp paired-read sequencing was performed on an Illumina HiSeq 2500 sequencer at Novogene Bioinformatics Technology Co., Ltd. (Beijing, China). The sequencing statistics of the RNA-seq were shown in Table S2.

10.1128/msystems.00534-22.2 TABLE S2 Summary of RNA-Seq data. Download Table S2, XLSX file, 0.01 MB. Copyright © 2022 Wang et al. 2022 Wang et al. https://creativecommons.org/licenses/by/4.0/ This content is distributed under the terms of the Creative Commons Attribution 4.0 International license.

### Transcriptome analysis.

Raw paired-end RNA-seq reads were quality filtered using Trimmomatic 0.33 (86). High-quality reads were mapped to the genome of *C. lactatifermentans* LBM19010 (CP046051) using BowTie2 2.2.6 (87). Gene expression levels were quantified by FeatureCounts and normalized as transcripts per million (TPM) (88). Gene expression results from FeatureCounts were used as input for the R bio-conductor package DESeq2 to perform differential expression analysis (89). Genes were considered differentially expressed if they had a |log_2_foldchange|>1 and adjusted *P*-value (padj) < 0.05. Upregulated and downregulated genes were categorized into COGs by using the information from the eggNOG database (90). COG enrichment analysis was performed by comparing the number of genes assigned to a given COG in a group of differentially expressed genes compared to the number of genes assigned to the same COG within the genome. Then, the hypergeometric test by R was used to calculate the enrichment probabilities with a statistical significance level of *P < *0.05.

### Fermentation validation.

To explore the effects of reducing power availability on caproate production, varying ratios of reducing substrate to acetate were used to culture *C. lactatifermentans*. With the same glucose equivalent of around 80 mM, seven different molar ratios of lactate to acetate and glucose to acetate were set up: 16:0 (only substrate), 15:1, 5:1, 1:1, 1:5, 1:15, and 0:16 (only acetate). Level 8 was set as the control group without reducing substrate and acetate supplemented.

To determine the effects of glucose on lactate utilization of *C. lactatifermentans*, we cultured this bacterium in mCGM supplemented with around 90 mM glucose as the carbon source and transferred the culture every 48 h. After 20 transfers, *C. lactatifermentans* was cultured in mCGM supplemented with 22 mM glucose and 102 mM lactate as carbon sources. Subsequently, the primary culture was transferred into a fresh mCGM supplemented with glucose and lactate as carbon sources at 72 h and 120 h, respectively. Fermentation broth samples (2.0 mL) were collected every 12 h until the end of the fermentation to determine the growth, substrate consumption, and metabolite production. All the fermentation experiments were performed in three biological replicates.

### Reverse transcription (RT)-PCR.

RNA from *C. lactatifermentans* cells grown in glucose medium was used to conduct RT-PCR for cotranscriptional analysis. cDNA was synthesized with genomic DNA-free RNA (1 μg) according to the manufacturer’s instruction of the BeyoRT III cDNA synthesis kit (Beyotime, Shanghai, China). The genomic DNA of *C. lactatifermentans* was used as the positive control and RNA (gDNA-free) was used as the negative control. Primers used for RT-PCR are listed in Table S3.

10.1128/msystems.00534-22.3 TABLE S3 List of primers used in this study. Download Table S3, XLSX file, 0.02 MB. Copyright © 2022 Wang et al. 2022 Wang et al. https://creativecommons.org/licenses/by/4.0/ This content is distributed under the terms of the Creative Commons Attribution 4.0 International license.

### Quantitative reverse transcription-PCR.

16 genes in center metabolic pathways were selected to validate RNA-seq data. The primers used for quantitative reverse transcription-PCR (qRT-PCR) are listed in Table S3. qRT-PCR was performed on the StepOnePlus real-time PCR system (Applied Biosystems, Foster City, CA). The relative expression level for each gene was calculated with the 2^-ΔΔCT^ method and normalized to the expression level of the 16S rRNA gene (91, 92).

### Analytical methods.

Acetate, butyrate, and caproate were analyzed by using gas chromatography (Agilent 7890B GC; Agilent Technology, Folsom, CA) equipped with a CP-Wax column (50 m × 0.25 mm × 0.25 μm; J&W Scientific, Folsom, CA) and a flame ionization detector (FID). The fermentation broth samples were centrifuged at 13,000 × *g* for 2 min. A 50 μL internal standard solution (pivalic acid, 12.5 g · L^−1^, pH 2.5) was added into 200 μL supernatant. After vortexing, 0.5 μL solution was then injected into the gas chromatograph with a split ratio of 30:1. The initial oven temperature was set to 60°C, maintained for 0.5 min, raised to 190°C at 20°C · min^−1^, and finally held at 190°C for 3 min. The temperature of the FID and the injection port was 220°C. Nitrogen was used as carrier gas at a flow rate of 2 mL · min^−1^. The hydrogen flow rate was 40 mL · min^−1^, and the airflow rate was 400 mL · min^−1^. The concentration of glucose and lactate were determined by a high-performance liquid chromatography system (Agilent 1200 HPLC; Agilent Technology, Santa Clara, CA) equipped with an Aminex HPX-87H column (300 × 7.8 mm; Bio-Rad Laboratories Inc., Hercules, CA) and a refractive index detector (RID). The column temperature was kept at 60°C. H_2_SO_4_ (5 mmol·L^−1^) was used as the mobile phase at a flow rate of 0.60 mL · min^−1^.

### Data availability.

Raw transcriptome reads have been deposited into the National Center for Biotechnology Information (NCBI) Sequence Read Archive (SRA) under the project accession number PRJNA841265.

10.1128/msystems.00534-22.10 DATA SET S1 RNA-seq data of *Caproicibacterium lactatifermentans* grown on glucose and lactate. Download Data Set S1, XLSX file, 0.3 MB. Copyright © 2022 Wang et al. 2022 Wang et al. https://creativecommons.org/licenses/by/4.0/ This content is distributed under the terms of the Creative Commons Attribution 4.0 International license.
