# Supplementary figures and images for: Revealing the Characteristics of Glucose- and Lactate-Based Chain Elongation for Caproate Production by Caproicibacterium lactatifermentans through Transcriptomic, Bioenergetic, and Regulatory Analyses
Source: mSystems. 2022 Sep 8;7(5):e00534-22. doi: 10.1128/msystems.00534-22 (PMC9600882; doi:10.1128/msystems.00534-22)

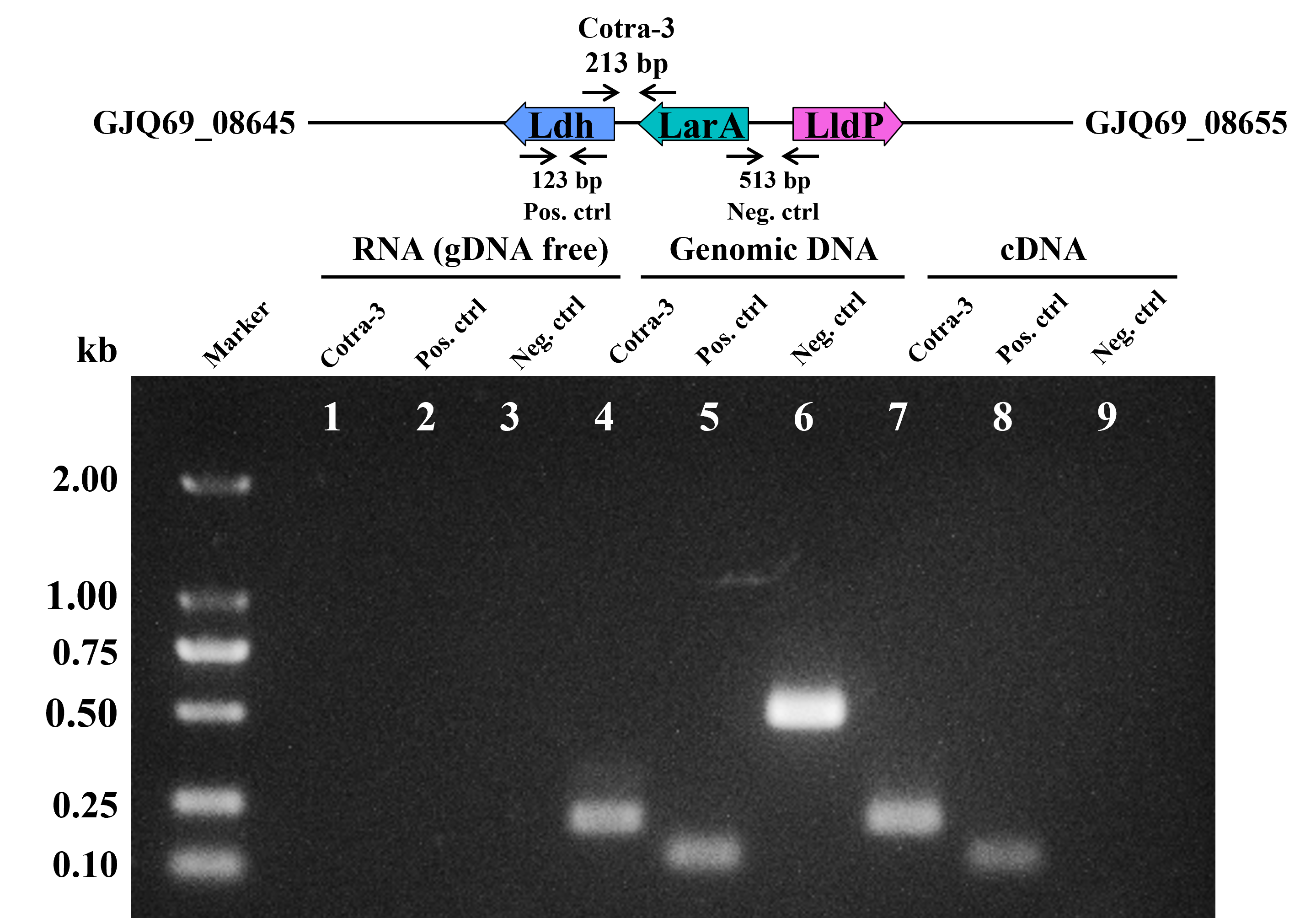

Supplement: FIG S6 [file msystems.00534-22-s0007.tif]
